# Central Contrast Sensitivity as an Outcome Measure in Randomized Controlled Trials in Glaucoma—A Systematic Review

**DOI:** 10.3390/life15071043

**Published:** 2025-06-30

**Authors:** Alexander Sverstad, Jens Riis Møller, Gianni Virgili, Augusto Azuara-Blanco, Josefine Freiberg, Simone Ahrensberg, Olav Kristianslund, Goran Petrovski, Miriam Kolko

**Affiliations:** 1 Center for Eye Research and Innovative Diagnostics, Department of Ophthalmology, Oslo University Hospital and Institute of Clinical Medicine, Faculty of Medicine, University of Oslo, 0450 Oslo, Norway; alexsvers@gmail.com (A.S.); olav.kristianslund@medisin.uio.no (O.K.); 2 Department of Drug Design and Pharmacology, University of Copenhagen, 2100 Copenhagen, Denmark; jensriismoeller@gmail.com (J.R.M.); josefine.freiberg@sund.ku.dk (J.F.); moneaa.a@gmail.com (S.A.); 3 Centre for Public Health, Queen’s University of Belfast, Belfast BT12 6BA, UK; g.virgili@qub.ac.uk (G.V.); a.azuara-blanco@qub.ac.uk (A.A.-B.); 4 Department of NEUROFARBA, University of Florence, 50139 Florence, Italy; 5 Department of Ophthalmology, University Hospital Centre, University of Split School of Medicine, 21000 Split, Croatia; 6 UKLO Network, University St. Kliment Ohridski, 7000 Bitola, North Macedonia; 7 Department of Ophthalmology, Copenhagen University Hospital, Rigshospitalet, Glostrup, 2600 Copenhagen, Denmark

**Keywords:** central contrast sensitivity, contrast sensitivity tests, glaucoma, glaucoma diagnosis, randomized controlled trials, systematic review

## Abstract

**Purpose**: Standard automated perimetry (SAP) remains the gold standard functional test in glaucoma, used primarily for evaluating peripheral vision loss. Central contrast sensitivity (CCS) has emerged as a potential early functional marker of glaucomatous damage. This systematic review aimed to describe the different methods used to measure CCS in randomized controlled trials (RCT) involving glaucoma patients. **Methods**: We searched the MEDLINE, Embase, CINAHL, Cochrane Central Register of Controlled Trials, Epistemonikos, and ClinicalTrials.gov databases on 25 January 2023, and updated the search on 12 February 2025. Eligible studies comprised RCTs that reported CCS as an outcome in patients with glaucoma, suspected glaucoma, or ocular hypertension. No restrictions were placed on age, sex, ethnicity, geography, intervention, or publication year. Abstracts and full texts were screened independently by two reviewers. Descriptive statistics were used. No formal risk of bias assessment was performed, due to the descriptive nature of the review. **Results**: Of 1066 records screened, 31 studies met the eligibility criteria. The study sample size ranged from 7 to 207 (median: 23), with most studies involving primary open-angle glaucoma. Interventions were diverse, mainly involving topical medications, with timolol being the most frequent. Eleven CCS test methods were identified. Five studies did not report the method used. The CSV-1000 was the most commonly used test, being applied in 11 studies. **Conclusions**: CCS has been measured using a wide range of methods in glaucoma RCTs, with limited standardization. Most of the included studies were small, variably reported, and conducted over 10 years ago, suggesting a decreasing interest in CCS as an outcome measure in glaucoma RCTs. **Funding:** This review was funded by Oslo University Hospital and the Research Council of Norway. **Registration:** This review was registered on the OSF.

## 1. Introduction

Glaucoma is an irreversible, chronic, progressive neuropathy of the optic nerve. If left untreated, it will lead to atrophy of the optic nerve, due to the death of retinal ganglion cells (RGCs) and their axons, ultimately resulting in reduced visual function and, eventually, blindness [1,2]. Glaucoma is traditionally considered a peripheral disease, often sparing the central vision until its later stages. However, this is not entirely accurate, as many cases of glaucoma present with defects within the central 10 degrees of vision [3,4]. To date, standard automated perimetry (SAP) is the gold standard functional exam for diagnosing and evaluating glaucoma progression. In the early stages of the disease, only the loss of RGCs and their axons can be detected, a stage referred to as pre-perimetric glaucoma [5,6,7]. A substantial loss of RGCs is needed before statistically significant visual field abnormalities can be seen [8,9,10], making early diagnosis challenging. Given that early intervention is critical in preventing visual impairment, improved diagnostic methods are needed.

Visual acuity (VA) is routinely performed in the evaluation of glaucoma patients but provides no useful information about the stage or progression of the disease. Despite maintaining good, best-corrected visual acuity (BCVA), many patients report decreased quality of vision. Studies suggest that central contrast sensitivity (CCS) and visual field loss correlate more strongly with self-reported visual function than VA [11,12].

CCS declines with age and is affected by various ophthalmic conditions and diseases [13]. This is also true for the early stages of glaucoma, where CCS has been widely used as an outcome measure in clinical trials [14,15,16,17,18]. However, contrast perception is a complex process influenced by multiple factors, including luminance conditions, target size, shape, orientation, color profile, and the distribution of spatial frequencies. CS is assessed both spatially and temporally. Spatial CS refers to the ability to discern differences in light and dark areas in static images, while temporal CS involves detecting changes over time, such as a flickering light. Numerous methods exist for assessing CCS, ranging from chart-based tests to computerized solutions, using a variety of stimuli, including optotypes, symbols, and black-and-white gratings [19]. Optotypes, while simple to use, are complex two-dimensional images that inherently contain a range of spatial frequencies and are, therefore, unsuitable for investigating specific spatial frequencies. Other tests standardize targets using black-and-white gratings with predefined spatial frequencies, measured in cycles per degree (cpd). Lower spatial frequencies correspond to wider stripes, while higher spatial frequencies correspond to narrower stripes. Humans typically have peak sensitivity to contrast at medium spatial frequencies (around 3–6 cpd) and decreased sensitivity at very low and very high frequencies [20].

To our knowledge, no systematic review has yet been conducted describing the use of CCS in randomized controlled trials (RCT) in glaucoma. It is important to summarize the existing evidence to inform the use of these tests in future research and clinical practice. In this context, we investigated whether they are used as primary or secondary outcomes, the types of tests used, data formats, and other study characteristics.

## 2. Methods

We registered the protocol of our systematic review in the Open Science Framework (osf.io) before data collection. The systematic review was assigned a registration DOI of https://doi.org/10.17605/OSF.IO/BWKQR.

### 2.1. Search Strategy

A systematic search was conducted on 25 January 2023, by a senior librarian at the Library of Medicine and Science at the University of Oslo, of the following scientific databases: MEDLINE (Ovid), Embase (Ovid), Cochrane Reviews and the Central Register of Controlled Trials, CINAHL (EbscoHost), Epistemonikos, Scopus, and ClinicalTrials.gov. The complete documentation of the literature search can be found in the Supplementary Materials.

**Figure 1:**
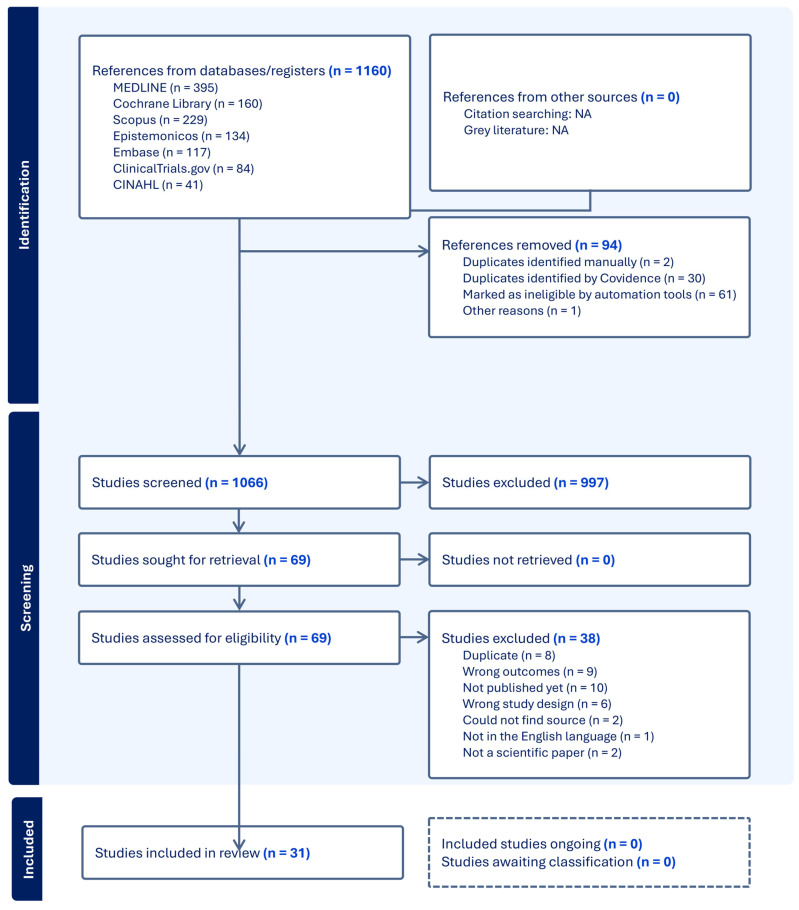
PRISMA flow diagram of the included and excluded studies.

An updated literature search was conducted on 12 February 2025, focusing on the three most relevant and comprehensive databases (MEDLINE, Scopus, and Epistemonikos). This targeted update approach was chosen to optimize time and resources while minimizing the risk of missing new relevant records. Although other databases were not re-searched during the update, we recognize this as a potential limitation. The same inclusion and exclusion criteria were applied. This update identified 79 additional records after duplicates were removed, of which 2 met the inclusion criteria and were incorporated into the final analysis. Both searches are summarized in the PRISMA diagram (Figure 1). 

### 2.2. Inclusion and Exclusion Criteria

We included glaucoma RCTs that used CCS as an outcome measure, with either temporal or spatial contrast sensitivity (CS). We included only publications in English but put no restrictions on the date, type of glaucoma (including suspected glaucoma and ocular hypertension), and severity. There were no limitations on age, sex, ethnicity, or geographical location. Any type of intervention and comparator was included. Duplicates, unpublished articles, and non-scientific papers were also excluded.

Abstracts were included only when they clearly described an RCT involving glaucoma patients and reported contrast sensitivity as an outcome. Given the limited number of eligible full-text studies, this approach allowed us to broaden the descriptive scope of CCS measurement methods. However, we acknowledge that abstracts provide less methodological detail, and this represents a limitation of our review.

### 2.3. Data Extraction and Quality Assessment

Two reviewers performed independent title and abstract screening processes, full-text reviews, and data extraction of the included studies. In the case of any uncertainty or disagreements, a third reviewer was consulted to reach a consensus. The review management software “Covidence” was used to organize the screening and extraction processes.

Data were retrieved from different sources, such as tables and text, and for studies only reporting their results with graphs, the data were visually assessed individually by each reviewer using either Autodesk Fusion 360 or Microsoft PowerPoint. Only descriptive statistics were used to summarize the data.

We extracted the following information from each study: the number of participants, intervention(s), type and description of the CCS test used, outcome format (e.g., log contrast, SPARCS score, and area under the contrast sensitivity curve), and follow-up duration. We recorded whether CCS was reported as a primary or secondary outcome, to assess how central it was to each study’s design. This distinction provides context on whether CCS was likely pre-specified and prioritized during planning or if it was included more exploratorily. CCS was considered to be a primary outcome when stated as such in the paper or if “contrast sensitivity” was mentioned in the title. In cases where relevant data were missing or unclear, we attempted to contact the corresponding authors for clarification. If no response was received or if the information could not be obtained, the item was recorded as “not reported” (NR).

Because this was a descriptive systematic review without statistical synthesis, we did not conduct a formal quality or risk of bias assessment. Our focus was on cataloguing the diversity of methods and outcome reporting, rather than comparing effect sizes or drawing pooled conclusions.

## 3. Results

### 3.1. Characteristics of the Included Studies

The PRISMA flow diagram of the included and excluded studies, along with their characteristics, is shown in Figure 1. The literature search resulted in a total of 1066 studies after the removal of duplicates and ineligible studies using the automation tool in Covidence. Of these studies, 997 were excluded after title and abstract screening. Full-text screening was performed on the remaining 69 studies, from which a further 38 articles were excluded. Reasons for their exclusion can be seen in the PRISMA flowchart (Figure 1). Therefore, a total of 22 published full-text papers and 9 abstracts were included in this review. These 31 articles and abstracts were published between 1990 and 2024. One of the records (Casson 2014 [21]) included a follow-up study, making the total number of studies 32. A summary of the 32 included studies can be seen in Table 1.

### 3.2. Reporting of CCS as a Primary or Secondary Outcome

Among the 32 studies (31 records) included, 8 specified CCS as a primary outcome, while only 1 study reported it as a secondary outcome (Table 1). The status of CCS as an outcome remained unspecified in the other 22 studies.

### 3.3. Follow-Up Time

Most of the included studies focused on the short-term effects on CCS. The most common follow-up time was one month (n = 14). Only 11 out of 32 studies had a follow-up time greater than one month (Table 1). Several studies used a crossover design, where follow-up duration can be interpreted in two ways: either as the total study duration or as the duration of each individual treatment period. In this review, we have chosen to report the duration of each treatment arm in the crossover studies, as this reflects the total exposure time for each intervention.

### 3.4. Types of Contrast Sensitivity Tests Used

To assess changes in CCS, the studies included a variety of different tests for CCS (Table 1). The most commonly used test to assess CCS was the CSV-1000 (n = 11). This test utilizes sine wave gratings of four different cpd of 3, 6, 12, and 18, arranged in rows. Each row has eight contrast levels, decreasing from left to right. The test presents a sample patch and eight pairs of gratings, together with a blank patch. The patient is asked to identify the patch with the gratings. The CCS is measured in both linear and log units.

Three studies used the Functional Acuity Contrast Test (FACT) chart. FACT utilizes 5 different cpd: 1.5, 3, 6, 12, and 18. In the same way as the CSV-1000, the gratings are presented in patches that decrease in contrast from left to right. FACT differs from the CVS-1000 by also tilting the patches 15° to either the left or right. The change in CCS is also measured in log units.

Despite being a well-known test for CCS, the Pelli–Robson chart was used in only three of the reviewed studies. In contrast to the other tests listed, the Pelli–Robson charts utilize Sloan letters instead of gratings. The letters are of equal size and have varying contrast levels. There are eight rows of letters, each consisting of two triplets. The change in contrast from one triplet to another is 0.15 log units, starting from the upper left, with 100% contrast (Weber contrast), to the lower right, with 0.56% contrast. The cpd of the Pelli–Robson chart depends on the test distance and how one measures the cpd, but it is, nevertheless, in the lower range and has been reported to be 1 [52] and 1.3 [53]) at 1 m.

Besides the three above-mentioned tests for CCS, the reviewed articles used a variety of different tests. These are summarized in Table 2, which provides a brief description of each test and its key characteristics.

### 3.5. Format of Data Outcome

Of the 31 studies, 16 used the logarithm of contrast sensitivity (log con); 12 studies did not provide any information about the data format, while 2 studies reported an area under the contrast sensitivity function (CSF) curve, 1 of which also showed the CSF curve with CS values. The authors of the final study reported their results as SPARCS scores.

### 3.6. Study Participants

Most of the included studies had a sample size below 50 (n = 24). The remaining eight studies had sample sizes ranging from 54 to 207 (Table 1).

### 3.7. Type of Glaucoma

The majority of the included studies investigated the effects of treatment on CCS in individuals with primary open-angle glaucoma (POAG) (n = 16). Other glaucoma subgroups investigated were normal tension glaucoma (n = 5), ocular hypertension (n = 2), and newly diagnosed POAG (n = 2). Open-angle glaucoma and primary angle-closure glaucoma were investigated in one study each, whereas another study included both types of glaucoma. The remaining three studies did not describe the type of glaucoma included.

### 3.8. Interventions

Several interventions were used in the included studies, with the most common treatments being eye drops of timolol (n = 10), latanoprost (n = 6), brimonidine (n = 6), and dorzolamide (n = 5). A wide selection of interventions was investigated using CCS, and the full list of interventions can be seen in Table 1. This included recognized glaucoma medications as well as treatments that are not normally associated with the treatment of glaucoma.

### 3.9. Types of RCTs Included

The reviewed articles showed a somewhat even distribution of placebo-controlled studies (n = 14) compared to studies investigating a comparative effect between two interventions (n = 18). The RCT design of the included studies was crossover (n = 15), parallel-group (n = 15), and split-body (n = 2) studies. The number and degree of blinding conducted were double (n = 19), single (n = 7), and none (n = 6).

## 4. Discussion

Our literature search identified eight studies that cited CCS as a primary outcome, with many not specifying its priority. The most common follow-up time was 1 month, but they varied between 15 min and 24 months. The sample size of the studies ranged from 7 to 207, averaging 37.5 participants. While POAG was the predominant glaucoma subtype examined, other subtypes were also considered. Common interventions included timolol, latanoprost, brimonidine, and dorzolamide eye drops, among a total of 23 different interventions being assessed for their impact on CCS. Across the 32 studies, 11 distinct CCS test methods were used, with the CSV-1000 and FACT emerging as the most frequently employed techniques.

While CCS testing has significant potential in detecting central visual impairment [13], its role in diagnosing and monitoring glaucoma remains uncertain. The findings suggest a decreasing interest in evaluating CCS in RCTs involving patients with glaucoma. This may, in part, be due to a lack of standardized methodologies, making comparisons across studies challenging. Unlike VA testing using the Snellen chart or visual field testing with SAP, CCS lacks a widely accepted gold-standard functional exam, limiting its adoption in clinical and research settings. Furthermore, most studies had short follow-up periods, which may fail to capture how different CCS measurement methods track changes over time or reflect the long-term effects of various interventions.

Our review highlighted substantial variability in CCS testing methods, with no universally accepted standard for assessment. The CSV-1000 and FACT charts were the most widely used tests, reflecting a preference for instruments that measure CCS across multiple spatial frequencies, offering a more detailed profile of visual function. Despite its widespread recognition and ease of use, the Pelli–Robson chart was used in only three studies. This may reflect its limitation in terms of sampling only low spatial frequencies and its complex two-dimensional design, which also includes a broad distribution of spatial orientations. Additionally, some tests were poorly described or omitted entirely, further complicating study comparisons. Temporal CS, which measures the ability to detect flickering stimuli, is notably underrepresented in the included literature. Although a few studies included methods that are sensitive to temporal modulations, most trials did not assess this dimension. This is a notable gap, given that temporal CS may be more strongly affected in glaucoma due to the early involvement of the magnocellular pathway [17]. This indicates the need for more comprehensive, standardized testing protocols that can account for both the spatial and temporal dimensions of contrast sensitivity in future RCTs involving patients with glaucoma.

The predominance of short-term follow-up periods in most of the studies, with only 11 out of 32 having follow-up periods exceeding one month, may further contribute to the uncertainty surrounding the role of CCS in glaucoma research. Given the progressive nature of glaucoma, longer follow-up periods are essential for understanding the potential role of CCS in monitoring disease progression and treatment efficacy. Additionally, only one study included a healthy control group, making it difficult to differentiate disease-related changes in CCS from normal variations in the general population. Shorter follow-up times may increase the impact of the learning effect, skewing the data in favor of a positive outcome. An important measure to minimize the learning effect is to implement training before collecting data for analysis. Out of the 32 studies, only 6 explicitly reported training to minimize the learning effect. Another study by Bose (1992) [25] used different charts instead of training to minimize the learning effect.

Only nine studies explicitly stated whether CCS was a primary or secondary outcome. Although we did not examine the study protocols, this shows a lack of adherence to reporting guidelines such as the SPIRIT and CONSORT checklists [62]. Clear and transparent outcome reporting is essential for the proper interpretation of findings, avoiding post hoc changes such as outcome switching.

Moreover, CCS may be affected by other ocular conditions, such as cataracts, which are common in the glaucoma population. Klein et al. (2015) [17] demonstrated that cataracts significantly decrease CCS and may interfere with early glaucoma detection when spatial and temporal CS tests are used. Their findings highlight the necessity of controlling for cataracts in CCS studies. Only one of the included studies (Bose et al. 1992 [25]) tried to control for this variable.

Our study has several limitations. First, while we aimed to include only RCTs, this approach excluded those insights from observational and cross-sectional studies, which could have provided additional context on CCS assessment in glaucoma. Second, although we assessed whether CCS was reported as a primary or secondary outcome, we did not review the study protocols to determine whether these outcomes were pre-specified, thereby limiting our ability to evaluate potential selective reporting bias. Third, this review only included published studies, potentially contributing to publication bias, as studies with negative or inconclusive findings are less likely to be published. Lastly, due to the descriptive nature of this review, we did not perform a formal quality or risk of bias assessment.

To further clarify the role of CCS in evaluating glaucoma patients, future research should aim to establish standardized protocols for CCS assessment. This requires longer follow-up periods, the use of healthy control groups, and comparative studies evaluating different CCS measurement methodologies. Additionally, controlling for cataracts is essential, as their impact on CCS may confound any findings related to early glaucomatous changes. Although our review did not include a meta-analysis comparing CCS with other glaucoma metrics, such an analysis could yield valuable insights into its diagnostic and prognostic utility. A quantitative synthesis of CCS relative to other functional and structural measures may help define its role in glaucoma management and assess its potential as an early marker of disease progression.

## 5. Conclusions

Our findings expose the diverse methodologies employed in CCS measurement, revealing a lack of standardization across clinical trials. This variability hinders the direct comparability of outcomes, thereby impeding the consolidation of data into further insights. Notably, the CSV-1000 and FACT charts emerge as prevalent tools, suggesting a preference for more detailed testing protocols. The relatively few studies included in this systematic review, especially those from the last decade, suggest a declining interest in evaluating CCS in RCTs involving patients with glaucoma. Furthermore, this systematic review highlights the necessity for future research to incorporate control groups and longer follow-up periods to better understand how different CCS measurement methods track changes over time.

## Figures and Tables

**Table 1:** Summary RCTs evaluating CS outcomes in patients with glaucoma. For crossover studies, both the total follow-up duration and individual treatment arm durations (including washout if applicable) are reported.

Author, Year Publication Type RCT Type	N Patients (HC)	CCS Test Used	Outcome Reporting of CCS	Intervention	Last Follow-Up	Main Finding
Muermans, 1990 [22] Abstract Crossover	20	NR	Primary	Timolol eyedrops or Placebo eyedrops	2 test sessions and 14 d washout	Data did not show any drug effects on performance for temporal modulation fields and spatial CS.
Balazsi, 1991 [23] Abstract, Crossover	15	NR	NR	Timolol eyedrops or Saline eyedrops	2 test sessions and 14 d washout	No significant effect of timolol on visual fields, temporal modulation, or CS in treatment-naive patients.
Bonomi, 1992 [24] Published paper Parallel group	20	Nicolet CS2000	NR	Dapiprazole 0.5% x 3 or Placebo	6 mo	No change in visual or contrast sensitivity in either group.
Bose, 1992 [25] Published paper Crossover	14 [17]	Pelli-Robson chart and Vistech near-contrast sensitivity chart	NR	Nimodipine capsules 60 mg once or Placebo once	2 h per arm 3 d washout	Contrast sensitivity was reduced in NTG eyes and improved following nimodipine treatment.
Drance, 1998 [26] Published paper Parallel group	68	Custom setup	NR	Betatoxolol 0.5% x 2 or Timolol 0.5% x 2 or Pilocarpine 2% x 4	24 mo	No significant differences in contrast sensitivity between groups.
Evans, 1999 [27] Published paper Crossover	11	CSV-1000E	NR	Betaxolol 0.25% x 2 or Timolol 0.5% x 2	4 mo in total: 1 mo per arm 1 mo washout	CS improved with both drugs, but only improved significantly with betaxolol at 6 cpd.
Harris, 1999 [28] Published paper Parallel group	29	CSV-1000	NR	Dorzolamide 2% x 3 or Placebo eyedrops	1 mo	Dorzolamide improved contrast sensitivity at low spatial frequencies in NTG patients.
Caramazza, 1999 [29] Abstract Parallel group	60	NR	NR	Polyunsaturated fatty acids (PUFA)	3 mo	Significant improvement in visual field and CS was observed after 3 months of PUFA treatment.
Garzozi, 2001 [30] Abstract Crossover	21	NR	NR	Latanoprost 0.005% x 1 Dorzolamide 2% x 3	1 mo per arm Washout NR	Neither drug altered the contrast sensitivity.
Sponsel, 2002 [31] Published paper Split-body	25	Two-step positive forced-choice algorithm (NeuroScientific)	NR	Unoprostone 0.15% x 2 or Latanoprost 0.005% x 1 + placebo	1 mo	CS increased with both drugs, with no difference between latanoprost and unoprostone.
Sponsel, 2002 [32] Published paper Hybrid split-body	20	Two-step positive forced-choice algorithm (NeuroScientific)	NR	Latanoprost 0.005% x 1 + placebo or Brimonidine 0.2% x 2 With/without Indomethacin po. 25mg x 4	1 mo	Both drugs improved CS, but the brimonidine group showed greater variability.
Arend, 2003 [33] Published paper Crossover	14	CSV-1000	NR	Timolol 0.5% x 2 or Dorzolamide 2% x 3 or Latanoprost 0.005% x 1	3 mo total 1 mo per arm	No significant change in CS after drug treatment.
Evans, 2003 [34] Published paper Parallel group	16	CSV-1000E	NR	Timolol 0.5% x 1 + placebo or Brimonidine 0.2% x 2	3 mo	Brimonidine improved CS. The effect may reflect neuroprotection rather than IOP reduction.
Harris, 2003 [35] Published paper Parallel group	20	NR	NR	Dorzolamide 2% x 3 or Latanoprost 0.005% x 1	1 mo	No conclusions regarding contrast sensitivity were drawn.
Catoira, 2004 [36] Abstract Crossover	31	CSV-1000	NR	Timolol 0.5% + Dorzolamide 2% x 2 or Brimonidine 0.2%	1 mo Details NR	A lower IOP was associated with better visual function in POAG.
Fea, 2004 [37] Abstract Parallel group	30	MAV Professional	Primary	Cyticholine i.m	2 wk	Citicoline improved visual function and contrast sensitivity.
Siesky, 2004 [38] Abstract Crossover	15	CSV-1000	NR	Brimonidine 0.2% x 3 or Timolol 0.5% x 2 + placebo	4 mo total 2 mo per arm	Significant association found between CS and retrobulbar blood flow in POAG patients.
Siesky, 2006 [39] Published paper Crossover	16	CSV-1000	NR	Tomolol x 1 + Dorzolamide x 1 + Placebo x 1 or Timolol x 2 + Latanoprost	3 mo total 1 mo per arm 2 wk washout	No difference in CS between treatments.
Evans, 2008 [40] Published paper Crossover	20	CSV-1000E	Primary	Latanoprost 0.005% x 1 or Timolol 0.5% x 1	6 mo total 3 mo per arm No washout	Latanoprost significantly improved the central CS more than timolol in POAG patients.
Prata, 2009 [41] Published paper Parallel group	54	Functional Acuity Contrast Test (FACT)	NR	Timolol 0.5% or Brimonidine 0.2% or Travoprost 0.004%	1 mo	Visual quality, VF MD, and high-frequency CS improved with treatment; no link to IOP or differences between drugs.
Casson, 2014 [21] Published paper Crossover	16 + 7 *	CSV-1000	Primary	50% topical glucose or Saline 0.9% 50% topical glucose or Saline 8%	2–3 wk total 15–30 min per arm 2–3 wk washout	Topical glucose significantly improved CS at 12 cpd; non-significant increases were seen at 3, 6, and 18 cpd.
Guo, 2014 [42] Published paper Crossover	35	Functional Acuity Contrast Test (FACT)	NR	Ginko biloba extract tablets or Placebo	4 mo total 1 mo per arm 2 mo washout	Ginkgo biloba showed no significant effect on CS compared to the placebo.
Aerie, 2019 [43] Published paper Parallel group	207	CSV-1000E	NR	Netarsudil solution of either 0.01%, 0.02%, or 0.04% or Placebo	1 mo	No conclusions were drawn with regard to CS.
Azizzadeh, 2019 [44] Published paper Parallel group	80	OPTEC Functional Vision Analyzer	Primary	Timolol 0.5% or Travoprost 0.004% or Dorzolamide 2% or Brimonidine 0.2%	15 min	Timolol and brimonidine caused temporary CS changes at 1.5, 3, and 18 cpd in POAG patients.
Trevino, 2019 [45] Abstract Parallel group	21	Pelli–Robson chart and CSV-1000	NR	LPI temporally OD + LPI superiorly OS or LPI superiorly OD + LPI temporally OS	1 mo	CS remained unchanged after superior or temporal LPI.
Marino, 2020 [46] Published paper Crossover	109	Spaeth/Richman contrast sensitivity test (SPARCS)	Primary	Citicoline 500 mg, homotaurine 50 mg, and vitamin E 12 mg (CIT/HOMO/VITE) +/− current topical treatment	8 mo total 4 mo per arm No washout	Preliminary data suggest that combined CIT/HOMO/VITE may enhance CS and vision-related quality of life in mild glaucoma.
Loughman, 2021 [47] Published paper Parallel group	62	OPTEC Functional Vision Analyzer	Secondary	Capsules containing 10 mg lutein, 10 mg meso-zeaxanthin, and 2mg zeaxanthin	18 mo	The study found an improvement in glare-affected mesopic contrast sensitivity.
Lee, 2021 [48] Published paper Crossover	38	Functional Acuity Contrast Test (FACT) chart in OPTEC 6500P	NR	Korean Red Ginseng 1.0 g x 3/day or Placebo	4 mo total 1 mo per arm 2 mo washout	Red ginseng improved CS and ocular pain in glaucoma; the effect may relate to retinal perfusion, not dry eye.
Hunter, 2022 [49] Abstract Parallel group	54	Functional Vision Analyzer	Primary	10 mg lutein + 10 mg meso-zeaxanthin + 2 mg zeaxanthin or Placebo	18 mo	Higher MPOV was linked to better CS and faster PRT; carotenoid supplementation may improve visual function in OAG. Further work is required to determine its longer-term effects on visual function.
Kitnarong, 2023 [50] Published paper Parallel group	32	OPTEC Functional Vision Analyzer	Primary	BF-IOL (AcrySof) or UVB-IOL (Tecnis)	2 mo	Cataract surgery improved VA and CS in glaucoma patients; no CS difference between UVB-IOL and BF-IOL.
Cadena, 2024 [51] Published paper Parallel group	16	Pelli–Robson chart	Primary	Repetitive transorbital alternating current stimulation (rtACS)	6 wk	Significant VR-QoL improvements were observed with rtACS.

* This record included a follow-up study of 7 patients. HC—healthy controls, NR—not reported, MPOV—macular pigment optical volume, PRT—photo-stress recovery time, BF-IOL—blue-light-filtering intraocular lenses, UVB-IOL—ultraviolet-blocking intraocular lenses, VR-QoL—vision-related quality of life.

**Table 2:** Short description of each of the CCS tests obtained from the included literature.

Name of Test (Frequency %)	Description
CSV-1000 and CSV-1000E (33%)	A standalone device with several test plates that can be attached to its backlit screen. The recommended test distance is 8 ft/2.5 m. The background luminance is auto-calibrated to 85 cd/m^2^. It consists of four rows of gratings labeled A–D. Each row contains eight pairs of circular patches, one with a sine wave grating and the other being blank. The contrast gradually decreases from left to right. The rows differ according to their cpd from top to bottom, at 3, 6, 12, and 18, respectively. Additionally, there is a single, larger grating of high contrast at the start of each row that serves as a sample. The patient’s task is to identify the patch with the gratings. This test is conducted for all four cpd plates to produce a contrast sensitivity curve [54].
FACT chart (9%)	A printed chart with five rows of increasing cpd of 1.5, 3, 6, 12, and 18. Each row consists of nine sine-wave gratings of decreasing contrast, oriented either vertically or at 15 degrees to either the right or left. The patient’s task is to determine what orientation the gratings have. This is performed for all five spatial frequencies/rows to produce a contrast sensitivity curve. The recommended test distance is 3 m [55,56].
Pelli–Robson chart (9%)	A printed chart with Sloan letters. The letters are of equal size and have varying contrast levels. There are eight rows of letters, each consisting of two triplets. The change in contrast from one triplet to another is 0.15 log units, starting from the upper left, with 100% contrast (Weber contrast), to the lower right, with 0.56% contrast [57]. The cpd of the Pelli–Robson chart is a more complex matter, one that is perhaps without much meaning. Unlike gratings, which can be described according to a few parameters (orientation, contrast level, and cpd), letters are more complex and contain a variety of spatial frequencies, orientations, and contrast. Nevertheless, the size of the letters will indeed affect the range of spatial frequencies, and, in the literature, it has been reported to be in the lower range of 1 cpd [52] and 1.3 cpd [53] at 1 m.
OPTECH Functional Vision Analyser (9%)	A stand-alone electronic device with multiple visual function tests in the form of slides that can be inserted into the device. It uses the FACT chart for CS testing. The device makes it easier to control for luminance, due to the goggle-like interface in which the patient places their face for testing, minimizing light contamination from the environment. The device has two luminance options, day testing, with 85 cd/m^2^, and night testing, with 3.0 cd/m^2^. In addition, it includes a glare-testing function with various illumination levels (1, 10, 28, and 135 LUX) [58].
OPTEC 6500P (3%)	An older version of the OPTEC FVA
SPARCS test (3%)	The Spaeth/Richman contrast sensitivity test (SPARCS) is a web-based test. It assesses both central and peripheral contrast sensitivity using a regular computer display. The tested contrast ranges from 100% to 0.45% (0.0 to 2.35 Log con). The test is structured by dividing the display into five areas: one central and four peripheral areas, with one in each corner. During testing, one of the five areas will display a grating of 0.4 cpd for 0.3 s; the patient should then click on the area where he/she saw the grating displayed. The result is presented as a SPARCS score, which is calculated from the results of the five areas [59].
MAV Professional (3%)	The system is no longer in production, and we were unable to find any other information.
NeuroScientific (6%)	We could not find any information on this system, other than what was written in the publication in which it was used. The paper stated: “Central retinal contrast sensitivity (7-degree pattern, 1 and 4 cycles per degree, 15 reversals per second) was performed using a two-step positive forced choice algorithm (NeuroScientific 8010, New York, NY, USA).” Contacting the authors was unsuccessful.
VCTS 6000 (3%)	A printed chart for testing contrast sensitivity at a near distance. Very similar to the FACT chart, but it is smaller (17.5 × 14 cm) and is used at a distance of 40 cm [60].
Nicolet CS 2000 (3%)	We were unable to find reliable information on this system. Most studies using this system are from the 1980s.
Custom Setup (3%)	The custom setup was described as a stationary, phase-reversing, sine-wave gratings sinusoidal, flickered in counter-phase at 25 Hz and with cpd of 0, 2, 5, 8, and 10 [61].

## Supplementary Materials

The following supporting information can be downloaded at: https://www.mdpi.com/article/10.3390/life15071043/s1, Documentation of literature search.
