# Supplementary material for: Central Contrast Sensitivity as an Outcome Measure in Randomized Controlled Trials in Glaucoma—A Systematic Review
Source: Life (Basel). 2025 Jun 30;15(7):1043. doi: 10.3390/life15071043 (PMC12299499; doi:10.3390/life15071043)
Supplement: Supplementary file 1 [file life-15-01043-s001.zip › life-3632702-supplementary.pdf]

PRISMA 2020 flow diagram for new systematic reviews which included searches of databases and registers only

Documentation of literature search

Research question: **What is the use of contrast sensitivity as an outcome measure in RCTs on glaucoma?**

The following databases were searched:

**Database**	**Number of retrieved references**
MEDLINE (Ovid):	409 + 60 (2023-2025)
Embase (Ovid):	241
Cochrane reviews and Central Register of Controlled Trials:	268
Cinahl (EbscoHost):	77
Epistemonikos:	205 + 9 (2023-2025)
Scopus:	376 + 100 (2023-2025)
ClinicalTrials.gov:	85
Number of references before deduplication:	1661
Number of references after deduplication:	991 + 128 (2023-2025)

All searches were done 25 January 2023 and 13 February 2025 (Medline, Epistemonikos, Scopus) by Toril M. Hestnes, senior librarian, University of Oslo, Library of Medicine and Science

Search syntax:

**Ovid-databases**	
exp/	Exploded index term
/	After an index term indicates a subject heading were selected.
.ti,ab,kf.	Search for a term in title, abstract and author keywords
.kw.	= keyword heading
*	At the end of a term indicates that this term has been truncated, diet* retrieves both diet, diets, dietary.
Adj3	Search for two terms next to each other, in any order, up to 3 words in between.
**Cochrane Library**	
ti,ab,kw	Search for word in title, abstract or keyword
NEAR/3	Search for two terms next to each other, in any order, up to 3 words in between.
**Cinahl**	
TI, AB	Search for word in title, abstract or keyword
N3	Search for two terms next to each other, in any order, up to 3 words in between.
MH	= subject heading
MM	= subject heading, major concept
**Scopus**	
TITLE-ABS-KEY	Search for word in title, abstract or keyword
W/3	Search for two terms next to each other, in any order, up to 3 words in between.

PRISMA 2020 flow diagram for new systematic reviews which included searches of databases and registers only

Ovid MEDLINE(R) ALL 1946 to January 24, 2023

#	Searches	Results
1	exp Glaucoma/	58888
2	(glaucom* or buphthalmos or hydrophthalmos).ti,ab,kf.	70302
3	(OAG or POAG or OHT or NTG).ti,ab,kf.	11487
4	exp Ocular Hypertension/	61548
5	exp Intraocular Pressure/	42541
6	IOP.ti,ab,kf.	23029
7	((intraocular or intra-ocular or ocular) adj (hypertension* or tension* or pressur*)).ti,ab,kf.	44075
8	or/1-7	109394
9	exp Contrast Sensitivity/	10298
10	contrast sensitivit*.ti,ab,kf.	7781
11	central contrast*.ti,ab,kf.	48
12	(CS adj2 (test* or chart* or curve* or score* or scoring or level* or value* or measur* or examination* or assess* or function* or impairment* or blur* or outcome* or high* or low* or normal)).ti,ab,kf.	7880
13	or/9-12	21435
14	8 and 13	710
15	exp Randomized controlled trials as Topic/	163880
16	exp Randomized controlled trial/	586706
17	Randomization/	106901
18	exp Random allocation/	106901
19	exp Double blind method/	174099
20	Double-Blind Studies/	174099
21	exp Single blind method/	32438
22	Single-Blind Studies/	32438
23	exp Clinical trial/	960960
24	exp Clinical Trials as Topic/	379907
25	Controlled Clinical Trial/	95167
26	exp Controlled Clinical Trials as Topic/	169577
27	exp control groups/	1896
28	Control Group/	1896
29	(randomized controlled trial or controlled clinical trial or pragmatic clinical trial or equivalence trial or Clinical Trial, Phase III).pt.	680286
30	(random* or sham or placebo*).ti,ab,hw,kf.	1771240
31	(nonrandom* or non-random* or quasi-random* or quasirandom*).tw,hw,kf.	53152
32	drug therapy.fs.	2565894
33	((singl* or doubl* or treb* or tripl*) adj (blind* or mask*)).ti,ab,kf.	194565
34	exp Placebos/	39395
35	(rct* or randomized or randomly or trial or group or groups or cohort* or placebo*).ti,ab,kf.	5668687
36	(allocated adj2 random*).ti,ab,kf.	39055
37	allocated.ti,ab,hw.	81491
38	(control* adj3 (intervent* or study or studies or trial* or group*)).tw,kw,kf.	1207346

39	(controlled or comparative or comparison or cohort or case control or pretest or pre-test or posttest or post-test or time series or patient series or epidemiological stud* or experimental stud* or quasi-random* or quasirandom* or quasi-experiment* or quasiexperiment* or non-random* or nonrandom*).ti,ab,kf.	3449868
40	exp Cohort Studies/	2439143
41	((open label or open-label) adj5 (study or studies or trial*)).ti,ab,hw,kf.	43395
42	((equivalence or superiority or non-inferiority or noninferiority) adj3 (study or studies or trial*)).ti,ab,hw,kf.	11580
43	(pragmatic study or pragmatic studies).ti,ab,hw,kf.	571
44	((pragmatic or practical) adj3 trial*).ti,ab,hw,kf.	7420
45	((quasiexperimental or quasi-experimental) adj3 (study or studies or trial*)).ti,ab,hw,kf.	11543
46	(phase adj3 (III or "3") adj3 (study or studies or trial*)).ti,hw,kf.	34582
47	(time series or patient series or experimental stud*).ti,ab,hw,kf.	163511
48	or/15-47	10630475
49	14 and 48	457
50	exp animals/ or exp animal experimentation/ or exp animal experiment/ or exp models animal/ or nonhuman/ or exp vertebrate/ or exp vertebrates/ or exp Cadaver/	26104845
51	exp humans/ or exp human experimentation/ or exp human experiment/	21015773
52	50 not 51	5089700
53	(veterinary or animal or animals or cadaver* or rabbit or rabbits or rodent or rodents or rat or rats or mouse or mice or rabbit or rabbits or pig or pigs or porcine or pigeon* or horse* or equine or cow or cows or cattle or bovine or goat or goats or donkey* or sheep or ovine or dog or dogs or canine or feline or beetle* or dolphin* or whale or whales or fish or fishes or zebrafish* or bluefish*).ti.	2424300
54	52 or 53	5579413
55	49 not 54	448
56	limit 55 to english	409
57	limit 56 to yr="2023 -Current"	60

Embase Classic+Embase 1947 to 2023 January 24

#	Searches	Results
1	exp glaucoma/	110633
2	(glaucom* or buphthalmos or hydrophthalmos).ti,ab,kf.	95409
3	(OAG or POAG or OHT or NTG).ti,kf.	906
4	exp intraocular pressure/	74071
5	exp intraocular pressure abnormality/	23324
6	exp intraocular hypertension/	13561
7	((intraocular or intra-ocular or ocular) adj (hypertension* or tension* or pressur*)).ti,ab,kf.	59343
8	IOP.ti,kf.	1950
9	or/1-8	162840
10	exp contrast sensitivity/	12333
11	contrast sensitivit*.ti,ab,kf.	10010
12	central contrast*.ti,ab,kf.	63
13	(CS adj2 (test* or chart* or curve* or score* or scoring or level* or value* or measur* or examination* or assess* or function* or impairment* or blur* or outcome* or high* or low* or normal)).ti,ab,kf.	12437
14	or/10-13	26776
15	9 and 14	1143

16	limit 15 to (randomized controlled trial or controlled clinical trial or phase 3 clinical trial)	91
17	exp randomized controlled trial/	762662
18	"Randomized Controlled Trial (topic)"/	246332
19	exp Controlled Clinical Trial/	954315
20	"Controlled Clinical Trial (topic)"/	13270
21	Randomization/	97873
22	Random Allocation/	94002
23	Double-Blind Method/	182301
24	Double Blind Procedure/	207198
25	Double-Blind Studies/	164320
26	Single-Blind Method/	47671
27	Single Blind Procedure/	49738
28	Single-Blind Studies/	49738
29	Placebos/	349296
30	Placebo/	406073
31	Control Group/	123692
32	(random* or sham or placebo*).ti,ab,hw,kf.	2490042
33	((singl* or doubl*) adj (blind* or dumm* or mask*)).ti,ab,hw,kf.	358584
34	((tripl* or trebl*) adj (blind* or dumm* or mask*)).ti,ab,hw,kf.	2007
35	(control* adj3 (study or studies or trial* or group*)).ti,ab,kf.	1689289
36	(nonrandom* or non random* or non-random* or quasi-random* or quasirandom*).ti,ab,hw,kf.	68139
37	allocated.ti,ab,hw.	105764
38	((open label or open-label) adj5 (study or studies or trial*)).ti,ab,hw,kf.	83592
39	((equivalence or superiority or non-inferiority or noninferiority) adj3 (study or studies or trial*)).ti,ab,hw,kf.	17396
40	(pragmatic study or pragmatic studies).ti,ab,hw,kf.	856
41	((pragmatic or practical) adj3 trial*).ti,ab,hw,kf.	8194
42	((quasiexperimental or quasi-experimental) adj3 (study or studies or trial*)).ti,ab,hw,kf.	18238
43	(phase adj3 (III or "3") adj3 (study or studies or trial*)).ti,hw,kf.	121456
44	or/17-43	3697132
45	15 and 44	260
46	16 or 45	260
47	(exp animal/ or exp animal model/ or nonhuman/) not exp human/	7821682
48	(veterinary or animal or animals or cadaver* or rabbit or rabbits or rodent or rodents or rat or rats or mouse or mice or rabbit or rabbits or pig or pigs or porcine or pigeon* or horse* or equine or cow or cows or cattle or bovine or goat or goats or donkey* or sheep or ovine or dog or dogs or canine or feline or dolphin* or whale or whales or beetle* or fish or fishes or zebrafish* or bluefish*).ti.	2949290
49	47 or 48	8278673
50	46 not 49	253
51	limit 50 to english language	241

Cochrane Library (Wiley)

#1 MeSH descriptor: [Glaucoma] explode all trees 3488
#2 (glaucom* or buphthalmos or hydrophthalmos):ti,ab,kw (Word variations have been searched) 8673
#3 (OAG or POAG or OHT or NTG):ti,ab,kw (Word variations have been searched) 1879
#4 MeSH descriptor: [Ocular Hypertension] explode all trees 3829
#5 MeSH descriptor: [Intraocular Pressure] explode all trees 3559

PRISMA 2020 flow diagram for new systematic reviews which included searches of databases and registers only

#6 (IOP):ti,ab,kw (Word variations have been searched) 6278
#7 ((intraocular or intra-ocular or ocular) next (hypertension* or tension* or pressur*)):ti,ab,kw (Word variations have been searched) 10334
#8 {OR #1-#7} 14087
#9 MeSH descriptor: [Contrast Sensitivity] explode all trees 686
#10 (contrast sensitivit*):ti,ab,kw (Word variations have been searched) 5425
#11 (central contrast*):ti,ab,kw (Word variations have been searched) 2777
#12 (CS near/2 (test* or chart* or curve* or score* or scoring or level* or value* or measur* or examination* or assess* or function* or impairment* or blur* or outcome* or high* or low* or normal)):ti,ab,kw (Word variations have been searched) 1124
#13 {OR #9-#12} 8730
#14 #8 and #13 268

CINAHL (EbscoHost)

S1 (MH "Glaucoma+")
9,901
S2 TI (glaucom* or buphthalmos or hydrophthalmos) OR AB (glaucom* or buphthalmos or hydrophthalmos)
9,559
S3 TI (OAG or POAG or OHT or NTG) OR AB (OAG or POAG or OHT or NTG) OR MW (OAG or POAG or OHT or NTG)
1,311
S4 (MH "Intraocular Pressure")
6,022
S5 TI ((intra*ocular OR ocular) N3 (hypertension* OR tension* OR pressur*)) OR AB ((intra*ocular OR ocular) N3 (hypertension* OR tension* OR pressur*))
4,953
S6 TI IOP OR AB IOP OR MW IOP
3,374
S7 (MH "Ocular Hypertension+")
10,200
S8 S1 OR S2 OR S3 OR S4 OR S5 OR S6 OR S7
16,156
S9 TI contrast sensitivit* OR AB contrast sensitivit*
1,621
S10 TI central contrast* OR AB central contrast*
301
S11 TI (CS N2 (test* or chart* or curve* or score* or scoring or level* or value* or measur* or examination* or assess* or function* or impairment* or blur* or outcome* or high* or low* or normal)) OR AB (CS N2 (test* or chart* or curve* or score* or scoring or level* or value* or measur* or examination* or assess* or function* or impairment* or blur* or outcome* or high* or low* or normal)) OR MW (CS N2 (test* or chart* or curve* or score* or scoring or level* or value* or measur* or examination* or assess* or function* or impairment* or blur* or outcome* or high* or low* or normal))
2,097
S12 S9 OR S10 OR S11
3,912
S13 S8 AND S12 Limiters - English Language
77

Epistemonikos

(title:((title:(glaucom* OR buphthalmos OR hydrophthalmos OR OAG OR POAG OR OHT OR IOP OR "intraocular hypertension" OR "intra-ocular hypertension" OR "ocular hypertension" OR "intraocular pressure" OR "intra-ocular

PRISMA 2020 flow diagram for new systematic reviews which included searches of databases and registers only

pressure" OR "ocular pressure") OR abstract:(glaucom* OR buphthalmos OR hydrophthalmos OR OAG OR POAG OR OHT OR IOP OR "intraocular hypertension" OR "intra-ocular hypertension" OR "ocular hypertension" OR "intraocular pressure" OR "intra-ocular pressure" OR "ocular pressure")) AND (title:(contrast sensitivit* OR central contrast* OR CS) OR abstract:(contrast sensitivit* OR central contrast* OR CS))) OR abstract:((title:(glaucom* OR buphthalmos OR hydrophthalmos OR OAG OR POAG OR OHT OR IOP OR "intraocular hypertension" OR "intra-ocular hypertension" OR "ocular hypertension" OR "intraocular pressure" OR "intra-ocular pressure" OR "ocular pressure") OR abstract:(glaucom* OR buphthalmos OR hydrophthalmos OR OAG OR POAG OR OHT OR IOP OR "intraocular hypertension" OR "intra-ocular hypertension" OR "ocular hypertension" OR "intraocular pressure" OR "intra-ocular pressure" OR "ocular pressure")) AND (title:(contrast sensitivit* OR central contrast* OR CS) OR abstract:(contrast sensitivit* OR central contrast* OR CS))))

Scopus

(((TITLE-ABS-KEY (random* W/4 (allocat* OR distribut* OR assign*))) OR (TITLE-ABS-KEY ({RCT} OR {RCT’s} OR {clinical trial} OR {controlled trial} OR {clinical-trial} OR {controlled-trial} OR {randomized} OR {randomised} OR {randomization} OR {randomisation} OR {randomly} OR {placebo} OR {trial} OR {groups} OR {subgroups}))) AND (((TITLE-ABS-KEY ({contrast sensitivity} OR {contrast sensitivities} OR {central contrast})) OR (TITLE-ABS-KEY (cs W/2 (test* OR chart* OR curve* OR score* OR scoring OR level* OR value* OR measur* OR examination* OR assess* OR function* OR impairment* OR blur* OR outcome* OR high* OR low* OR normal)))) AND ((TITLE-ABS-KEY ({intraocular hypertension} OR {intra-ocular hypertension} OR {ocular hypertension} OR {intraocular pressure} OR {intra-ocular pressure} OR {ocular pressure} OR {IOP})) OR (TITLE-ABS-KEY ({glaucoma} OR {glaucom} OR {glaucomas} OR {glaucomatous} OR {buphthalmos} OR {hydrophthalmos} OR {OAG} OR {POAG} OR {OHT} OR {NTG}))))) AND NOT (TITLE (veterinary OR animal OR animals OR rabbit OR rabbits OR rodent OR rodents OR rat OR rats OR mouse OR mice OR rabbit OR rabbits OR pig OR pigs OR porcine OR pigeon* OR horse* OR equine OR cow OR cows OR cattle OR bovine OR goat OR goats OR donkey* OR sheep OR ovine OR dog OR dogs OR canine OR feline OR dolphin* OR whale OR whales OR beetle* OR fish OR fishes OR zebrafish* OR bluefish* OR seabass)) AND (LIMIT-TO (LANGUAGE , "English"))

ClinicalTrials.gov

contrast sensitivity OR central contrast OR CS | Interventional Studies | glaucoma OR glaucomas OR OAG OR POAG OR IOP OR intraocular hypertension OR intra-ocular hypertension OR ocular hypertension OR intraocular pressure OR intra-ocular pressure OR ocular pressure

OR

contrast sensitivity OR central contrast OR CS | Observational Studies | glaucoma OR glaucomas OR OAG OR POAG OR IOP OR intraocular hypertension OR intra-ocular hypertension OR ocular hypertension OR intraocular pressure OR intra-ocular pressure OR ocular pressure

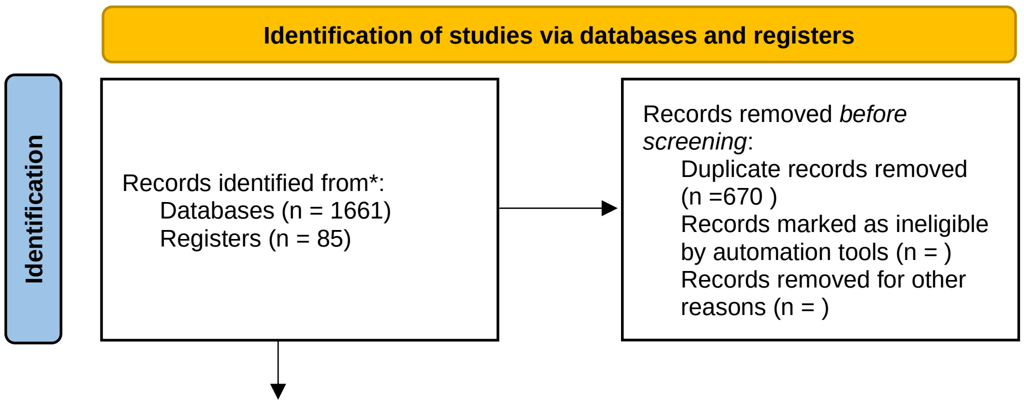

PRISMA 2020 flow diagram for new systematic reviews which included searches of databases and registers only

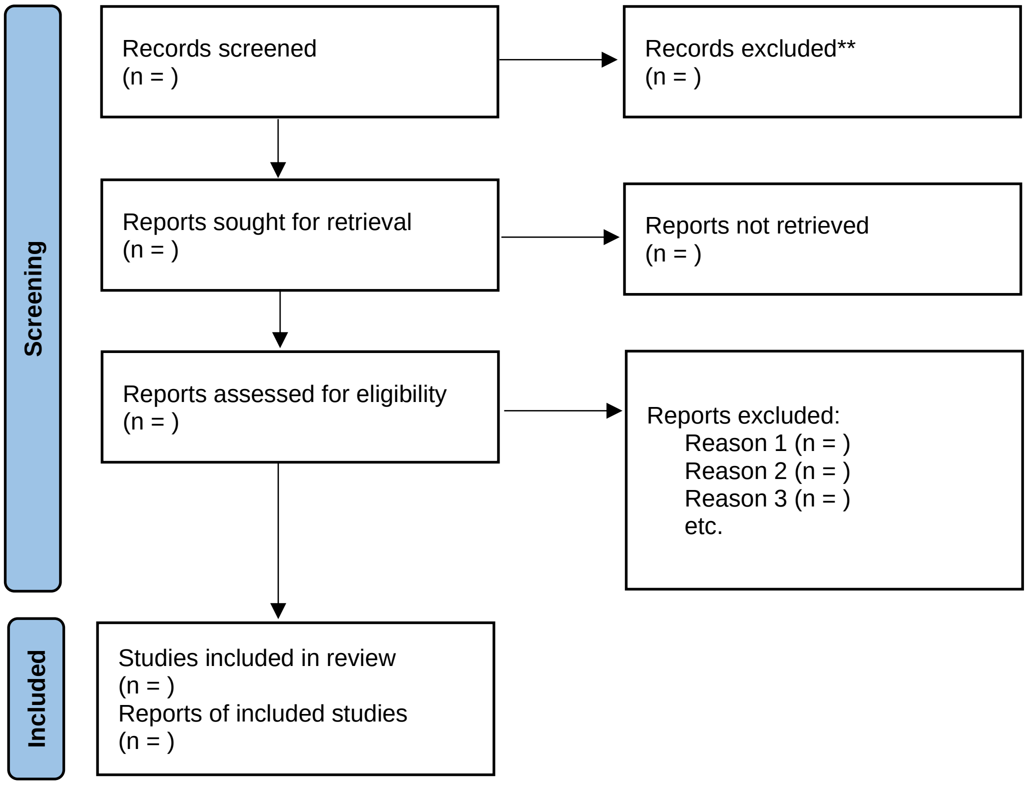

*Consider, if feasible to do so, reporting the number of records identified from each database or register searched (rather than the total number across all databases/registers).

**If automation tools were used, indicate how many records were excluded by a human and how many were excluded by automation tools.

From: Page MJ, McKenzie JE, Bossuyt PM, Boutron I, Hoffmann TC, Mulrow CD, et al. The PRISMA 2020 statement: an updated guideline for reporting systematic reviews. BMJ 2021;372:n71. doi: 10.1136/bmj.n71

For more information, visit: http://www.prisma-statement.org/

PRISMA 2020 flow diagram for new systematic reviews which included searches of databases and registers only
